# Prevalence and antibiotic resistance patterns of diarrheagenic *Escherichia coli* isolated from adolescents and adults in Hamedan, Western Iran

**Published:** 2013-03

**Authors:** Mohammad Yousef Alikhani, Seyyed Hamid Hashemi, Mohammad Mehdi Aslani, Safar Farajnia

**Affiliations:** 1 Department of Microbiology, Faculty of Medicine, Hamadan University of Medical Sciences, Hamedan, Iran; 2 Department of Infectious diseases, Faculty of Medicine, Hamedan University of Medical Sciences, Hamedan, Iran; 3 Department of Microbiology, Pasteur Institute of Iran, Tehran,Iran; 4 Biotechnology Research Center, Tabriz University of Medical Sciences, Tabriz, Iran

**Keywords:** Diarrhea, *Escherichia coli*, Antimicrobial resistance, Hamadan

## Abstract

**Background and Objectives:**

Pathogenic strains of *Escherichia coli* are a common cause of acute infectious diarrhea. The aim of this study was to investigate the frequency, virulence markers and antibiotic resistance patterns of diarrheagenic *E. coli* (DEC) isolated from adolescents and adults in Hamadan, west of Iran.

**Materials and Methods:**

A total of 187 stool samples were collected from adults with acute diarrhea. Stool culture was performed by conventional methods for enteropathogenic bacteria. Virulence factor genes for DEC were detected by polymerase chain reaction. Antimicrobial susceptibility was tested using the disk diffusion method.

**Results:**

Among the 187 patients, 40 (21.4%) were positive for DEC. The most frequently identified DEC was enteropathogenic *E. coli* (47.5%), followed by enteroaggregative (20%), enterotoxigenic (17.5%) and shiga-toxin producing *E. coli* (15%). No isolates of enteroinvasive *E. coli* were detected. All STEC strains were *stx*+ / *eae*A-. Out of the seven ETEC strains, five (71.4%) produced ST, one (14.3%) produced only LT and one (14.3%) of the isolates produced both ST and LT encoded by est and elt genes, respectively. Among the 40 DEC strains 27(67.5%) were multidrug resistant.

**Conclusion:**

DEC contribute to the burden of diarrhea in adults in Hamadan. Enteropathogenic *E. coli* was the most commonly identified DEC strain in the region studied.

## INTRODUCTION

Infectious diarrheal diseases are a major cause of morbidity and mortality in developing countries. In the past decades several new enteric pathogens, including bacterial, viral, and parasitic agents have been described. However, since most of the burdens of diarrheal diseases are incurred among infants and children in developing countries, the majority of diarrhea etiology studies are performed on children in such regions (1–7).

Pathogenic strains of *Escherichia coli* are a common cause of acute infectious diarrhea. *E. coli* can cause diarrhea by different mechanisms. Each type of *E. coli* diarrhea is associated with a different pathotype of *E. coli*, and each pathotype has characteristic virulence determinants that contribute to its pathogenic mechanisms (8). Five pathotypes of diarrheagenic *E. coli* (DEC) which have been identified in different countries include: enterotoxigenic *E. coli* (ETEC), Shiga toxin–producing *E. coli* (STEC), enteropathogenic *E. coli* (EPEC), enteroinvasive *E. coli* (EIEC), and enteroaggregative *E. coli* (EAEC). Many types of STEC and EPEC have the *eae* chromosomal gene, encoding the outer membrane protein intimin, and both strains elicit attaching and effacing lesions on the intestinal mucosa (9). EPEC and STEC are distinguished by the presence of the Shiga toxin–encoding gene, being present only in STEC. ETEC is a common cause of traveler's diarrhea (10) which is caused by the production of heat-labile (LT) and/or heat-stable (ST) enterotoxins. EIEC is similar to *Shigella* species by means of genetic traits of virulence. EAEC have been associated with acute and persistent diarrhea in children and adults in developed and developing areas (11, 12).

Numerous studies have reported the role of diarrheagenic *E. coli* in childhood diarrhea. However, only a few reports have involved the etiologic role of *E. coli* pathotypes in teenagers and adults’ diarrhea (13). Of important notice is the development of multidrug resistance in all pathotypes of *E. coli* isolated from children with diarrhea (14, 15). In the last decades, resistance against the commonly used drugs for the treatment of enteric infections including ampicillin, tetracycline, and co-trimoxasole has increased among DEC (16). This resistance in the developing countries has been attributed to the extensive use of antibiotics and poor prescription practices. This problem may also affect the isolates from adults with diarrhea.

The aim of this study was to determine the frequency of diarrheagenic *E. coli* strains in the teenagers and adults’ diarrheal diseases by polymerase chain reaction (PCR) technique and antibiotic resistance patterns.

## MATERIALS AND METHODS

During July 2008-July 2009, a total of 187 stool specimens were collected, from adolescents and adults with acute diarrhea who attended the outpatient clinic of Sina Hospital in Hamadan. Among the 187 samples, 112(59.9%) were from males and 75(40.1%) from female patients. The age range of patients was from 12 to 65 years (with a mean age of 39.3 years). Diarrhea was defined as a history of more than one liquid stool per day or three or more stools of loose consistency during the previous 24 hours.

### Microbiologic methods

Fresh stool specimens obtained from patients were inoculated into the Cary- Blair transport medium (BD BBL™, USA), which was refrigerated on the day of collection and transported within two days to the microbiology laboratory of Hamadan University of Medical Sciences. All the specimens were cultured for pathogenic bacteria using standard methods (17). Three individual lactose-fermenting and 2 non-lactose-fermenting colonies from MacConkey medium were pooled and subjected to direct PCR for *E. coli* virulence genes (*elt, est, eae,ial*, and *stx and pCVD432 plasmid)*.

### Polymerase chain reaction assays

The PCR for diarrheagenic *E. coli* was performed in patients whose stools grew *E. coli*. To detect STEC (*stx* and/or *eae* genes), ETEC (*elt* and *est* genes), EIEC (*ial* gene), EPEC (*eae* gene), and EAEC (EAEC-associated plasmid *p*CVD432) PCR assays were performed using the primer pairs listed in Table 1, (18–22). DNA was isolated from whole organisms by boiling method (23). Bacteria were harvested from an overnight broth culture, suspended in 1 mL of sterile distilled water, and incubated at 100°C for 10 minutes. Following centrifugation of the lysate, 1 μL (approximately 50 ng DNA) of the supernatant was used in the PCRs. Diarrheagenic *E. coli* reference strains EIEC/ Shigella ATCC 43893 (*ial* ^+^), EPEC 2348/69 (*eaeA* ^+^), EAEC E-17-2 (*pCVD432* ^+^), ETEC ATCC 35401 (*elt* ^+^ and/or, *est* ^+^), STEC EDL933 (*stx* ^+^ and/or *eaeA* ^+^) were used as positive controls and *E. coli* K12 (ATCC 10798) as negative control.

**Table 1:** Polymerase chain reaction (PCR) primers.

Pathogen	Gene target	PCR primers	Size of product (basepairs)	Reference
**EPEC**	*eae*	5'-TTATggAACggCAgAggT-3' 5'-CTTCTgCgTACTgCgTTCA-3'	790	13
**ETEC**	*elt*	5'-TCTATgTgCATACggAgC-3' 5'-ATACTgATTgCCgCAAT-3'	322	14
	*est*	5'-TAAACAAgTAgAggTCTTCAAAA-3' 5'-CggTACAgAgCAggATTACAACA-3'	147	14
**STEC**	*stx*	5'-ACgAAATAATTTATATgT-3' 5'-TgATTgTTACAgTCAT-3'	900	15
**EAEC**	*pCVD432*	5'-ggCgAAAgACTgTATCAT-3' 5'-ATgTATAgAAATCCgCTgTT-3'	630	16
**EIEC**	*ial*	5^'^-CTggTAggTATgCTgAgg-3' 5'-CCAggCCAACAATTATTTCC-3'	320	17

ETEC (enterotoxigenic *Escherichia coli*); EAEC (enteroaggregative *E. coli*); EPEC (enteropathogenic *E. coli*); EIEC (enteroinvasive *E. coli*). Attaching and effacing locus (*ea*e); Heat-labile toxin (LT)**;** Heat-stable toxin (ST); Shiga toxin (stx); EAEC-associated plasmid pCVD432; Invasion-associated locus (*ial*).

### Antibiotic susceptibility testing

Antimicrobial susceptibility testing was performed by the disk diffusion method according to the Clinical and Laboratory Standards Institute guidelines (24). Antimicrobial agents tested were ampicillin (10 µg), tetracycline (30 µg), co-trimoxazole (25 µg), chloramphenicol (30µg), cefotaxime (30 µg), ceftriaxone (30 µg), cefexime (5 µg), gentamycin (10 µg), nalidixic acid (30 µg), ciprofloxacin (5 µg), and norfloxacin (10 µg) (HiMedia Laboratories Co., India). The *E. coli* strain ATCC 25922 was included as a quality control in all assays.

### Statistical analysis

Data were analyzed using SPSS version 11 software. Percentages were compared using a Pearson chi-square test for dependent samples or Fisher's exact test when appropriate.

## RESULTS

Out of the 187 patients, 81(43.3%) had positive stool culture including 40(21.4%) *E. coli*, 26(14%) *Shigella*, 7(3.7%) *Salmonella*, 6(3.2%) *Klebsiella*, 1(0.5%) *Yersinia enterocolitica*, 1(0.5%) *Campylobacter jejuni*. As shown in Table 2, EPEC was the most prevalent (47.5%) DEC isolated in patients, followed by EAEC, ETEC and STEC. Isolates of EIEC were not detected. All the STEC strains were *stx*+/*eae*A-. Out of the 7 ETEC strains, 5(71.4%) produced ST, 1(14.3 %) produced LT, and 1(14.3%) of the isolates produced both ST and LT, encoded by *est* and *elt* genes, respectively. Out of the 40 DEC strains, 19(47.5%) were recognized as EPEC, 8(20%) as EAEC, 7(17.5%) as ETEC and 6(15%) strains as STEC.

**Table 2:** Pathogenic species identified in the fecal samples positive for diarrheagenic *E. coli*.

Age groups	EPEC(*eae*+)	EAEC (*pcvd432*+)	ETEC(elt+/est)	STEC (*stx*+)	Total

		No(%)			
**< 20**	13 (72)	1(5.5)	0 (0)	4 (22)	18 (100)
**≥ 20**	6(27.2)	7(31.8)	7(31.8)	2(9)	22(100)
**Total**	19(47.5)	8(20)	7(17.5)	6(15)	40(21.4)

The antimicrobial susceptibility profile of the diarrheagenic *E. coli* to 11 antibiotics is shown in Table 3. DEC isolates were resistant to most of the antibiotics tested. Antimicrobial resistance patterns for patients who had DEC included 87.5% resistant to ampicillin and 75% to co-trimoxazole. Out of the 40 DEC strains 27(67.5%) were multidrug resistant (resistance to > 3 antimicrobial drugs), and the major resistance profile was ampicillin/ co-trimoxazole/tetracycline. Moreover, 29 (72.5%) isolates were resistant to tetracycline, 20(50%) to cefexim and cefotaxime, and 1(2.5%) to ciprofloxacin and norfloxacin. Comparison of resistance patterns between patients belonging to different DEC groups showed that STEC was less resistant to ampicillin and co-trimoxazole than ETEC, EPEC and EAEC. ETEC was significantly more resistant (p < 0.05) to ciprofloxacin and norfloxacin than other DEC groups.

**Table 3:** Antimicrobial resistance profile of diarrheagenic *E. coli* isolates.

DEC (n)	AMP	CTX	CRO	CFM	TET	GM	CL	SXT	NA	NOR	CIP

n (%)											
**EPEC(19)**	16(84.2)	14(73.6)	2(10.5)	2(10.5)	12(63.2)	2(10.5)	6(31.6)	14(73.7)	3(15.8)	0(0.0)	0(0.0)
**EAEC (8)**	8(100)	7(87.5)	1(12.5)	7(87.5)	7(87.5)	6(75)	4(50)	6(75)	2(25)	1(12.5)	1(12.5)
**ETEC (7)**	6(85.7)	5(71.4)	3(42.8)	2(28.6)	6(85.7)	2(28.6)	1(14.3)	5(71.4)	2(28.6)	2(28.6)	1(14.3)
**STEC (6)**	5(83)	4(66)	1(17)	4(67)	5(83)	1(17)	3(50)	4(67)	2(28.6)	0(0.0)	0(0.0)
**Total (40)**	35 (87.5)	30 (75)	7 (17.5)	15 (37.5)	30 (75)	11 (27.5)	14 (35)	29 (72.5)	9 (22.5)	3 (7.5)	2 (5)

AMP, ampicillin, CRO, ceftriaxone, CTX, cefotaxime, CFM, cefexime, TET, tetracycline, GM, gentamycin, CL, chloramphenicol, SXT, co-trimoxazole, NA, nalidixic acid, NOR, norfloxacin, CIP, ciprofloxacin

## DISCUSSION

The frequency, virulence markers and antibiotic resistance patterns of several pathotypes of DEC, not routinely studied in adults, has been described in this study. The molecular targets used in this study have been substantiated as important virulence factors for each of the organisms and/ or have been correlated with diarrhea in epidemiologic studies.

In this study, enteropathogenic bacteria were isolated from 43.3% of adolescents and adults with acute diarrhea. Previous studies indicate that bacteria are the ethiologic agents of 20% to 60% of the total diarrhea cases. Although viruses are the most common causes of acute diarrhea in children in industrial countries, bacteria are the most prevalent enteropathogens in children and adults in developing countries (13).

In this study, DEC was the most common isolated bacteria. DEC has been reported as the most frequently identified pathogen in various studies throughout the world. Some studies, however, have reported different bacteria as the leading entropathogen in adults, such as *Salmonella spp*. in Tunisia and New Caledonia (25, 26) and *Campylobacter jejuni* in Sweden (13). Moreover, in a study in Austria, no cases of DEC were diagnosed (27). Some of these regional differences may be related to study population or stool culture techniques. Indeed, in agreement with this study, DEC was frequently isolated in developing countries such as India (28), Nijeria (29), Thailand (30), and Korea (31).

The most common pathotype of DEC in the current study was EPEC, followed by EAEC. These two pathotypes are among the most common enteric pathogens isolated from children less than five years in developing countries (14, 15, 32). In adults, ETEC and EAEC are the most common causes of travelers’ diarrhea, respectively (33). In a large study of bacterial enteropathogens among hospitalized diarrhea patients in all age groups from India, EPEC was the most frequent DEC pathotype (28). On the other hand, a study of DEC infection in two regions in the United States implicated EAEC as the most common bacterial cause of diarrhea in all age groups (34). A large study in the United Kingdom also identified EAEC as the second most common bacterial enteropathogen after *Campylobacter* species (35). These studies indicate the important role of EAEC and EPEC in diarrhea both in children and adults.

In this study, no cases of infection with EIEC were diagnosed. Most other studies also rarely reported this pathotype in teenagers and adults. Only one study in recent years reported EIEC as the predominant diarrheagenic bacteria in Ecuador (36).

Most DEC strains isolated in this study were resistant to ampicillin and co-trimoxasole (87.5% and 75% resistance, respectively). Similar results reported in other studies indicate the universal resistance of DEC to these agents. Higher rates of resistance have been reported both in children and adults in recent years (14, 34, 35, 37).

In the current study, 67.5% of the DEC isolates were multidrug resistant (MDR). The most resistance pattern was ampicillin/co-trimoxasole/tetracycline. The incidence of diarrhea due to MDR *E. coli* has increased in developing countries in the last decade. Higher rates have been reported from 50 to 70% in recent years (11, 16, 38), and the highest rate up to 75% have been reported from India (38, 39). Selective antibiotic pressure associated with the inappropriate use of antibiotics may be responsible for the antimicrobial resistance. Also, dissemination of transferable plasmids encoding multidrug resistance has been observed in the DEC isolates from diarrheal stools and surface waters. Use of surface waters for drinking or irrigation raises an important hazard for dissemination of MDR DEC in the developing countries (40).

At present, because of increased frequency of MDR DEC, fluoroquinolones are considered as the first-line drugs for the treatment of diarrhea. Indeed, several studies have documented the emergence and spread of fluoroquinolone resistant enteric pathogens (28, 39) and thus, a monitoring of drug susceptibility of DEC seems to be a critical issue in Iran.

In conclusion, this study reveals that DEC strains contribute to the burden of adult diarrheal diseases and EPEC is the most commonly identified DEC strain in Hamedan. To stop the increasing prevalence rate of MDR DEC, the indiscriminate use of antibiotics needs to be avoided and the guidelines for proper use of antibiotics for the treatment of diarrhea in this region needs to be established.
